# Occurrence and Risk of Metal(loid)s in *Thelesperma megapotamicum* Tea Plant

**DOI:** 10.3390/plants9010021

**Published:** 2019-12-23

**Authors:** Christine Samuel-Nakamura, Felicia S. Hodge

**Affiliations:** 1 School of Nursing, University of California, Los Angeles (UCLA), 4-246 Factor Bldg., Mailcode 691821, Los Angeles, CA 90095, USA; 2 School of Public Health, UCLA, 5-940 Factor Bldg., Mailcode 691921, Los Angeles, CA 90095, USA; fhodge@sonnet.ucla.edu; 3 School of Nursing, UCLA, 5-940 Factor Bldg., Mailcode 691921, Los Angeles, CA 90095, USA

**Keywords:** tea, uranium, cadmium, molybdenum, Navajo, Diné, provisional tolerable weekly intake, tolerable upper limit, metal(loid)s, greenthread

## Abstract

This study reports on the harvesting, ingestion, and contamination of American Indian tea *Thelesperma megapotamicum* grown on the Navajo Reservation in New Mexico. Uranium (U) and co-metal(loid)s (As, Cd, Cs, Mo, Pb, Se, Th, and V) have contaminated local soil and plants. Tea plants were gathered for analysis near U mining impacted areas. The study collected samples of wild tea plants (*n* = 14), roots (*n* = 14), and soil (*n* = 12) that were analyzed with inductively coupled plasma mass spectrometry. Tea harvesting activities, behavior, and ingestion information were collected via questionnaires. Harvesting took place in community fields and near roadways. Results indicate edible foliage concentration levels for Cd exceeded the World Health Organization (WHO) raw medicinal plant permissible level guidelines. Tea samples collected near high traffic areas had significantly greater Cd and Mo concentrations than those collected near low traffic areas (*p* < 0.001). Tea sample metal(loid) concentration levels ranged from 0.019–7.916 mg/kg. When compared to established food guidelines including the WHO provisional tolerable weekly intake (PTWI), reference dietary intake, recommended dietary allowance, and the tolerable upper limit (UL), Cd exceeded the WHO guidelines but none exceeded the PTWI nor the UL. These findings warrant improved standardization and establishment of universal guidelines for metal(loid) intake in food.

## 1. Introduction

Worldwide tea is one of the most popular beverages, second only to water [1]. The top growers and harvesters of herbal teas are India, Asia, the Middle East, and South America [2]. The benefits of herbal tea are touted by many for their medicinal, stress relieving, and antioxidant properties. Herbal tea has been shown to improve weight loss [3] and maintenance [4,5,6] as well as reduce the risk of diabetes onset [5,6] and heart disease with resultant decreased low-density-lipoprotein cholesterol [7,8]. Moreover, many teas may possess antimicrobial [9] and anti-cancer effects [10,11].

American Indian (AI) Diné ethnobotanical uses of *Thelesperma megapotamicum* (Sprengel) Kuntze include its popular use as a beverage and diuretic, and its use as an agent to improve hematuria, pelvic pain, bladder stones, anuria [12], and indigestion [13]. The plant has several cultural uses including as a dye for wood, wool, and textiles (producing orange yellow or gold hues) [13]. Vernacular names include greenthread, Hopi or Navajo tea, and cota. Several additional *Thelesperma* species exist, each with specific uses such as the treatment of dental pain, nervous stimulant (*T. gracile*) [14], tea substitute (*T. longipes* and *T. trifidum*), and wood dye (*T. subnudum*). The local Diné names for the tea plant are “c’îl dehî” (“wild mountain tea”) [15] or “ch’il gohwééh” (plant coffee). *T. megapotamicum* species provide a significant portion of the food derived energy of the Diné diet [16] and serves as an important cultural resource. This plant species has a wide distribution and extends from the southwest plains of the United States to South America [17]. *T. megapotamicum* is utilized by many Indigenous and non-Indigenous communities in North and South America.

Herbal teas are popular, easily accessible, low-cost, and presumed to be safe. However, multiple studies have documented toxicity, adverse events, untoward side effects, reactions (such as overdose, tolerance, and dependence and/or addiction), hypersensitivity and allergic reactions, and mid to long-term effects on multiple organ systems (including the heart, nervous, liver, and renal systems), genotoxicity, and teratogenicity [18,19]. Traditional communities are known to rely on herbal plants for medicinal and other cultural resources, but these natural harvest sites tend to be contaminated by anthropogenic activities [20]. The Navajo Reservation where the Diné people reside has been heavily impacted by U mining, which has resulted in more than 550 abandoned mines and waste sites. The U mining era occurred from the 1940s to the 1980s [21]. Hard rock mining areas are a problem in this and other AI communities with more than 160,000 mining sites existing [21] in the western U.S. Mining impacted areas on Diné lands have a potential to effect food resources as demonstrated by several reports on harvested herbs, forage plants, crops, meat, soil and water [22,23,24].

International tea studies abound [19,25], however, few studies have examined AI tea harvesting, consumption, and uses. Even fewer studies have assessed AI tea harvested in environmentally contaminated mining areas where known contaminants were previously documented [23,26,27], with most plant studies primarily focusing on tea’s biomacromolecular fractionation [28] and its antioxidant properties [29,30].

Various contaminants in tea may pose health risks for those who consume it. Uranium is a renal chemical toxicant [31] and Cd is known to cause renal problems [32]. Lead causes neurodevelopmental problems [33]. Metalloids like As and Se are known teratogens [34,35], as are metals like Mo and Cs [36,37,38]. Thorium has been shown to cause cancer in high doses [39] and V can cause adverse respiratory effects [40]. Long-lasting and permanent deleterious health effects are common with metal(loids) such as As, Pb, Cd, and Se. Many factors and exposure pathways exist and include plant species, the environment and soil, and plant stages of harvesting such as its location, collection, and its manner of preparation. Determining these factors and pathways are important in identifying ways to mitigate dietary exposure factors and risks.

This study determined the metal (Cd, Cs, Pb, Mo, T, U, and V) and metalloid (As and Se) concentration levels in the tea plant, *Thelesperma megapotamicum* and soil sampled from within and near harvesting sites in known U mining impacted areas on Diné tribal lands in New Mexico (NM). The calculated human ingestion for the study cohort was reported in relation to the World Health Organization (WHO) raw medicinal plant permissible level (RMPPL); the provisional tolerable weekly intake (PTWI), the Food and Nutrition Board (FNB) reference dietary intake (RDI) or recommended dietary allowance (RDA), and tolerable upper limit (UL) for As, Cd, Pb, Mo, and Se. Notably, there are no established dietary guidelines for Cs, Th, or U. Based on a comparison between the WHO RMPPL and the PTWI guidelines, recommendations are presented.

## 2. Materials and Methods

### 2.1. Setting

**Figure 1:**
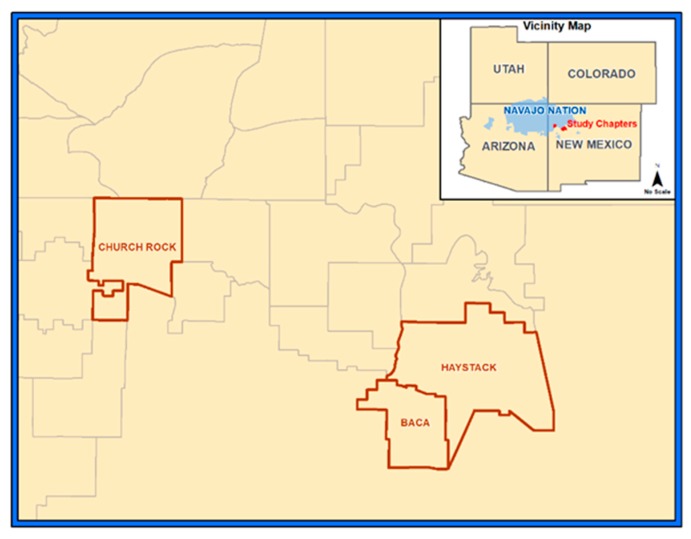
Participating chapters or communities (Churchrock and Baca/Haystack) on the Navajo Reservation in northwestern New Mexico. Inset shows their proximity to the Four Corners region of the USA.

Study samples were collected from August 1, 2012 to October 2, 2012 in a semi-arid region of the Navajo Reservation in northwestern NM (Figure 1). According to the monthly climatic meteorological data, the average precipitation is 25 cm per year [41]. The mean elevation is 2108 m (standard deviation [SD] = 43) and the mean temperature for the collection dates was 17.20 ± 1.89 °C. Two “chapters” or communities, participated in the study, with their total, collective land mass being 764 km^2^ (individual land sizes were 531 and 233 km^2^).

Using a descriptive comparative design, samples of live *T. megapotamicum* plants coupled with soil samples were collected and their concentration of metals (U, Cd, Cs, Pb, Mo, Th, and V) and metalloids (As, Se) were determined. Plant samples were collected near homes, in remote harvesting fields, and near local roadways.

The secured tribal community agreements permitted the administration of questionnaires and the collection of plants and soil specimens on reservation lands. Active consenting was conducted with a bilingual (Diné/English) language proficient researcher. The study was reviewed and approved by the University of California, Los Angeles (UCLA) Institutional Review Board (IRB# 11-001594-CR-00005) and the Navajo Nation Human Research Review Board (#NNR-11.321). *T. megapotamicum* is not listed as an endangered or threatened plant species according to the Navajo Nation Division of Natural Resources [42] or the state of NM Energy, Minerals, and Natural Resources Department [43].

Individuals recruited to participate were adult Diné residents of the included communities who were aged 18 years or older, not pregnant, were community residents for at least 10 years, and who harvested and consumed local foodstuffs. Participants recruited through flyers and meetings held at “Chapter Houses” (community meeting houses). Two questionnaires on harvesting practices, behavior, and dietary intake were administered to participating residents. Plant specimens were collected in the northwestern region of the Navajo Reservation where known contaminants were previously documented [26,27].

### 2.2. Herbal Tea Samples

Live tea samples were collected from wild, non-cultivated sources within a 3.2 km radius of the central part of abandoned U mines and features (mine portals, rim strips, pits, prospect areas, and vertical mine shafts). In addition, they were collected near vehicle roadways <0.8 km from high traffic areas (HTAs) and >2 km from low traffic areas (LTAs). The entire live plant (aboveground portions and roots) was stored in polyethylene (PE) plastic Ziplock^®^ bags. The samples were weighed, photographed, bagged, and placed on dry ice for shipment for analysis by the Analytical Chemistry Laboratory Earth and Planetary Sciences Department at the University of New Mexico (UNM). The stems, leaves, flowers, and roots of *T. megapotamicum* were analyzed for metals (Cd, Cs, Pb, Mo, T, U, and V) and metalloids (As, Se) using inductively coupled plasma-mass spectrometry (ICP-MS).

### 2.3. HTA and LTA Classifications

Traffic data were classified according to the New Mexico Department of Transportation [44] functional classification system. The classification was categorized by traffic road volume, speed, and number of lanes, from Grade 1 having the highest traffic volumes, speed, and lanes and Grade 7 having the lowest traffic volumes, speed, and lanes. For the current study, the HTA tea samples were collected near traffic ways <0.8 km and were classified as Grades 1 through 4. The mean distance between HTA tea samples and the edge of busy roadways was 0.64 km (SD = 0.6, range 0.58–0.71 km). Tea samples from LTAs were collected >2 km from roads that were classified as Grades 5 through 7.

### 2.4. Soil Samples

Parallel soil samples were collected for each plant. A silicon-coated-core sampler (Art’s Manufacturing and Supply Inc. (AMS), American Falls, ID, USA) was utilized to avoid cross contamination. A stainless-steel hand auger with a slide hammer was employed to collect soil samples using a PE liner (AMS Core Sampling Mini-kit, American Falls, ID, USA). One hundred grams (g) of soil were collected each from topsoil (0–15 cm depth) and root soil (5–25 cm depth). The soil samples were also analyzed for metal(loid)s (As, Cd, Cs, Pb, Mo, Se, Th, U, and V) using ICP-MS.

### 2.5. Human PTWI Calculation Equations

Human intake calculations were completed for As, Cd, and Pb [45,46] as follows:

PTWI = daily intake of metals = ∑[concentration of metals in tea x mean of tea intake (grams per person per day)], weekly intake of metals = daily intake x seven days week; weekly intake per body weight (kg) (PTWIs) = weekly intake reference body weight (60 kg).

The number of grams per day of tea consumed (5 g) for this study was based on comparative data [28,47].

### 2.6. Plant Identification and Nomenclature

Parallel live tea plant samples were collected and placed in a plant press until dry. They were identified and archived by the UNM Herbarium. The global positioning system (Trimble Navigation Limited, Westminster, CO, USA) was used to document the location of each plant, a description of the herb, and general county and state location information were collected.

### 2.7. Sample Analysis

Tea and environmental sample preparation and analysis have been reported in detail in previous publications [22,23,24]. Field samples were stored in a −20 °C freezer before sample preparation and analyses. The organic biota samples were oven dried at 65 °C. The plants were washed with 18 mega Ohm water then soaked in dilute 0.01 N HCl. The samples were prepared by weighing 2 g dry mass into the digestion tube. Two mL hydrogen peroxide (H_2_O_2_) and 5 mL of ultra-high purity (UHP) nitric acid (HNO_3_) were added and the solid samples (soil and plants) were gradually heated to 95 °C and digested for two hours. The digested samples were transferred into 50 mL volumetric flasks and brought to volume using 18 mega Ohm water. Three mL of HNO_3_ (reagent blank) was run with each batch of samples. PerkinElmer NexION 300D ICP-MS (Waltham, MA, USA) was utilized to analyze the samples. The method detection limits are as follows: As 0.3 μg/L, Cd 0.1 μg/L, Pb, 0.008 μg/L, Mo 0.02 μg/L, Se 1.3 μg/L, and U 0.008 μg/L. Three replicates were measured for each sample. Certified reference materials were used and include the National Institute of Standards and Technology (NIST) Standard Reference Material (SRM) 1573a (Tomato Leaves, NIST Gaithersburg, MD, USA) and 2709 San Joaquin Soil (NIST, Gaithersburg, MD, USA) producing the following values for Cd and V: Cd: 1.474 ± 0.11 mg/kg (certified reference tomato leaves: 1.52 ± 0.04 mg/kg) and V: 0.94 ± 0.07 mg/kg (certified reference tomato leaves: 0.84 ± 0.01 mg/kg) and Cd: 0.64 ± 0.09 mg/kg (certified reference soil value: 0.37 ± 0.02 mg/kg) and V: 83.2 ± 7.7 mg/kg (certified reference soil value: 110 ± 11 mg/kg). Satisfactory precision results were found with relative SDs ranging from 7.3% to 13.8%.

### 2.8. Statistical Analysis

Statistical Package for the Social Sciences (SPSS) for Windows (version 23, IBM, Armonk, NY, USA) was used for statistical analysis. Metal(loid) concentration levels in the tea and soil samples were reported as milligrams per kilogram (mg/kg). Summary data included means, standard deviations, medians, ranges, and percentages. Differences between metal(loid) levels in tea parts, soil, and HTA versus LTA were compared, with significance determined by the Student’s *t*-tests. A *p* value of <0.05 was considered significant. The absolute value of the *t*-statistic is reported, along with relevant means and/or interpretation of direction of difference.

## 3. Results and Discussion

### 3.1. Human Tea Harvester Data

All participating Diné tea harvesters were female. Their mean age was 61 years (SD = 10.1, range 43–78 years) and they had resided and utilized the local harvesting areas for a mean of 54 years (SD = 5.67). On average, tea users included in the study ingested local *T. megapotamicum* tea 1.54 days per week for a mean of 35 years (SD = 26, range 6–58 years). All participants boiled dry and fresh tea, utilized all aboveground portions of the tea plant (leaf, stem, and flowers), and had utilized both young and mature plants. The tea was not mixed or combined with other herbs or substances except infusion water. Two-thirds of the harvesters reported rinsing tea herbs before boiling and ingestion. All participants shared the tea free of charge with people residing off and on the reservation. Only one participant reported selling the tea both on and off Diné lands. One third of the tea harvesters had previously utilized *T. megapotamicum* as a pigment to dye sheep wool textiles.

Food harvesting behavior was determined to identify and document the various pathways of exposure. Researchers were able to demonstrate that participants had lived near the harvesting areas for extensive periods of time (> 5 decades) and had consumed tea for more than three decades. Tea harvest areas were used by multiple generations. In most instances, the tea harvesting areas had existed and were used prior, during, and after the peak mining era. In examining tea preparation behavior, this cohort exclusively infused the aboveground portion of tea plant while excluding the root portion. This was a positive finding, the majority of the tea roots contained greater metal(loid) concentrations than their aboveground counterparts. One-third of participants also reported not rinsing the raw tea before preparation and ingestion. This is another potential route of exposure that has been identified and poses opportunities for behavioral modification. A study by Jing et al. [6] demonstrated that rinsing tea leaves with distilled water removed 44% of Pb, which suggests a clear case for educational intervention. As with this study, food sharing behavior within the local community has been found to be extensive and common with other North AI communities [48]. The extent to which food is shared or sold with local harvesting communities bears further study, characterization, and provides educational opportunities. 

### 3.2. Metal(loid) Concentration Levels in Tea Plant Parts and Soil

The majority of the aboveground portions of tea had less metal(loid) concentrations than the roots (Table 1). The metal(loid)s that met statistical significance were As (t (26) = 4.84; *p* < 0.05), Cd (t (26) = 1.47; *p* < 0.05), Cs (t (26) = 3.03; *p* < 0.05), Mo (t (25) = 1.64; *p* < 0.05), Pb (t (26) = 6.40; *p* < 0.05), U (t (26) = 8.20; *p* < 0.05), and V (t (26) = 4.93; *p* < 0.05). This finding is consistent with other international tea studies [49,50]. In fact, it was reported that the feeding roots (vs. main roots) acted as a buffer for aboveground plant part Cd and As uptake [50]. Other studies have reported that low pH (5.5–6.5) can facilitate Cd transfer and accumulation [51,52]. The current study pH was 6.5 ± 0.6, which may have been an important factor in this study. Other important variables affecting metal(loid) uptake reported in the literature include geology, atmosphere, climatic conditions [53], co-occurrence with other metal(loid)s, physicochemical properties of the soil, exposure period, dispersion range, and uptake ability of plant species [20,54]. The contamination may occur at various tea harvesting stages and includes the environment from which the plant was harvested, along with the collection, preparation, and processing of the plant [28], steeping or brewing conditions, storage [53], and transport [55]. It is beyond the scope of this paper to describe all the variables associated with metal(loid) uptake in detail.

Topsoil metal(loid) concentration levels were greater than the root soil; although only Pb was found to be statistically significant (*p* < 0.05). Most metal(loid)s showed the following concentration distributions: root soil > topsoil > root > herb. However, the concentration distributions for Mo and Se were in the order of root > topsoil > herb. The greatest concentration differences occurred in the aboveground portions compared to the root for Mo (7.916 mg/kg vs. 18.304 mg/kg) and V (0.244 mg/kg vs. 2.343 mg/kg), with each differing by one order of magnitude. 

The overall U concentration levels were the lowest in all aboveground tea parts and root categories, except in root soil and topsoil (it was the third and fourth lowest in the latter categories). Generally, smaller U concentrations levels were demonstrated in squash crop, but greater metal(loid) concentrations (As, Pb, Se, and U) were seen in seven species of aboveground sheep forage plants, root, and soil in the parent study [22,24].

International studies have examined diverse tea species and found varying metal(loid) concentration levels. First, the current study found greater concentration levels for Cd, Mo, and V. Several studies have reported lower Cd concentrations in China (from below detection to 0.10 mg/kg) [50,56,57]. The Mo concentration levels in this study were greater than in other tea studies (0.04–4.2 mg/kg) [58,59]. Antal et al. [60] found lower concentrations than what was detected with V concentrations of 0.031–76.3 mg/kg in tea aerial parts and 0.026–14.5 mg/kg in tea leaves. Equivocal concentration levels were found with studies examining Cd, Pb, and Se studies. A tea study in Turkey found comparable Cd concentrations in tea leaves (0.05–1.27 mg/kg) [61]. The study concentration ranges for Pb in China and the Middle East were of similar range to the current study (0.30–3.42 mg/kg) [56,62,63]. Se concentrations fell between 1.12 to 2.26 mg/kg for Kolachi et al. [64] and from 0.096 to 0.114 mg/kg for Nookabkaew and associates [65]. Finally, several studies have determined and reported higher metalloid concentration levels for four metal(loid)s. The As concentration levels seen in our study were lower than those in Asia (0.024–1.53 mg/kg) [50,56,65], likewise Steenkamp et al. [66] reported higher ranges of U concentrations at 10–60 mg/kg in African herbal samples from a U mining impacted area. Two recent studies reported greater Th concentration levels ranging from 0.597 to 9.918 mg/kg [67] and from 2.46 to 14.76 mg/kg [68]. Cesium radioactivity concentration levels were reported by Oprea et al. [68] and Mitrovic et al. [69], however, the current study did not determine radiological risk.

The current study found that the Cd concentration level (0.35 mg/kg) in this popular species of tea herb exceeded the WHO RMPPL of 0.3 mg/kg [45,70]. Additionally, the WHO [71] recommends that medicinal plants used for various herbal formulations (teas, tinctures, etc.) should be evaluated for the presence of heavy metals [70]. The RMPPL of toxic metals are set at 1 mg/kg for As and 10 mg/kg for Pb [28,71]. 

### 3.3. Metal(loid) Concentration Levels in HTAs and LTAs

The mean distance between LTA *T. megapotamicum* samples was 6.01 km (SD = 4.40, range: 2.12–11.55). In the majority of samples, HTA metal(loid) concentration levels were greater (Cd, Pb, Mo, Se, Th, U, and V, *p* < 0.001) than those of LTAs (As and Cs, *p* < 0.05). However, only five metals were found to be statistically significant: Cd (t (12) = 15.18; *p* < 0.001), Mo (t (111) = −4.70; *p* < 0.001), U (t (12) = 0.74; *p* < 0.05), V (t (12) = 1.20; *p* < 0.05), and Cs (t (12) = 2.44; *p* < 0.05 (Table 2). 

Several studies found that tea collected closer to roadways contained significantly more contaminants [6,54,72]. More narrow distances (50–100 m) from HTAs also showed there were less contaminants associated with plants harvested for tea [72]. A comparison of the metal(loid) concentrations from this current study to the WHO RMPPL guidelines revealed unsafe levels of Cd, as high as 226% above the guideline for samples from HTAs. Fortunately, the As and Pb levels did not exceed the WHO RPMML guidelines. Roadway contaminants are known to result from anthropogenic activities such as mining, re-suspended road dust, water run-off, vehicle part wear, tire wear, and vehicular emissions [72,73]. Furthermore, HTAs pose an increased risk of metal inhalation, ingestion, and dermal exposure according to Steenkamp et al. [66]. 

### 3.4. Human PTWI Calculations

The weekly intake calculations for *T. megapotamicum* tea were 0.25 μg/kg for As, 0.20 μg/kg for Cd, and 0.18 μg/kg for Pb (Table 3). The PTWI percentages for the aboveground portion of tea were all less than 3% (1.7% for As, 2.9% for Cd, and 0.7% for Pb). The PTWIs for the current tea ingesting cohort were not exceeded.

In other parts of the world, the vast majority of tea studies did not exceed the PTWIs [28,49,74,75,76]. In some instances, the RMPPL or other guideline was used for reference. For example, Caldas and Machado’s [33] examination of several medicinal herbs exceeded the WHO RMPPL for Cd and Pb, with Pb in horse chestnuts (*Aesculus hippocastanum*) exceeding the PTWI by 440% (Cd did not exceed the PTWI). Similarly, Han et al. [49] found that Pb exceeded the national maximum permissible concentration (MPC), but not the PTWI. In the first case, the researchers recommended stricter systematic control on the use of medicinal plants (particularly for *A. hippocastanum*). In the second case, the investigators reported both guidelines and concluded that the contribution to human Pb intake from drinking tea was small, but considerable at the highest concentration. Neither study discussed the discrepancy between the various guidelines.

### 3.5. Human Intake Calculations for RDI or RDA and UL

The daily intake calculations were 8.7 μg for Mo, 0.8 μg for Se, and 0.3 μg for V (Table 4). The results showed that 19.4% of the RDA for Mo and 1.5% of the RDI for Se were consumed from tea. There is no established RDI or RDA for V [77]. The calculated intakes for V, Se, and Mo were all below the UL (0.02%, 0.2%, and 0.4 %, respectively). None of the ULs were exceeded for the listed metal(loid)s in any of the samples. Arsenic, Cd, Cs, Pb, Th, and U have no established RDIs, RDAs, or ULs. 

None of the FNB guidelines were exceeded in all categories. However, the micronutrients Mo and Se were below the required RDA and RDI, respectively. In this cohort, additional Se and Mo from foods may be necessary such as nuts, legumes (Mo) meats, and grains (Se). As such, the RDI and RDA requirements may have been met in this study sample by the combination of tea ingestion and other foods consumed, however, this study exclusively focused on one harvested food item of the entire diet. In the parent cohort, varying findings were found with Mo and Se whereby squash did not meet the Mo RDA and Se RDI [24]. However, both micronutrients were exceeded in sheep meat by 205% (Mo RDA) and 713% (Se RDI) [22]. It is advisable to consider all foods consumed collectively to avoid exceedances above the UL. The advisement of dietary intake from a health care provider or dietitian is recommended in similar settings.

### 3.6. Human PTWI Calculations for Samples from HTAs and LTAs

The HTA and LTA weekly intake calculations were, respectively, 0.19 and 0.17 μg/kg for Pb, 0.21 and 0.27 μg/kg for As, and 0.40 and 0.06 μg/kg for Cd (Table 5). All were below 6% of the PTWI. With the exception of As, all the HTA percentages were greater than the LTA percentages. For Cd, the difference between HTAs and LTAs was one order of magnitude. When considering the PTWIs collectively from Table 3, the percentages were lower (0.7–2.8%) than by a comparison of PTWI percentages by HTA and LTA (0.7–5.7%; Table 5), particularly for Cd with the HTA greater by nearly 5%. No recent literature has compared the PTWIs between HTAs and LTAs. However, Han et al. [63] completed a traffic study examining Pb concentration levels at incremental distances from the road from 0–200 m, however, the reported findings did not relate the distances to the calculated PTWIs.

### 3.7. Human Intake Calculation Estimates for RDI or RDA and UL for Samples from HTAs and LTAs

The daily intake calculations for HTAs and LTAs, respectively, were as follows: V was 0.3 μg and 0.2 μg, Se was 1.1 μg and 0.6 μg, and Mo 19.9 μg and 0.4 μg (Table 6). The differences in percentages between samples from HTAs and LTAs were greater for Mo and Se than V. The Mo RDA for HTA samples was 44% compared to 0.9% for the LTA samples. HTA samples contained 2.0% of the RDI for Se versus 1.1% for LTA samples. As indicated above, there are no established RDAs or RDIs for V. The calculated daily intakes for Mo, Se, and V were all below the ULs, ranging from 0.02% to 0.99% for HTA samples and from 0.01% to 0.2% for LTA samples. 

Mo had a difference between HTA and LTA samples of two orders of magnitude and reflectively the RDA for HTA was a large percentage higher (about 43%). Consuming tea from HTAs to meet the RDA for Mo is not recommended. Mo has a very limited number of studies focused on tea [70] and needs further characterization and research. Similar to the collective calculation of RDI or RDA and ULs, the HTA and LTA findings were below all guideline categories.

### 3.8. Differences between the WHO RMPPL and the PTWI Guidelines

*T. megapotamicum* tea and soil were examined and the human implications were examined by calculating the PTWI, RDA, or RDI and ULs. There were notably large differences found between whether or not Cd exceeded the threshold based on whether the WHO RMPPL or the PTWI was used for the guideline. In a 1989 WHO document [45], the RMPPL guidelines were shown to be based on the acceptable daily intake (ADI) values. The actual derivation of the WHO RMPPLs is not shown. According to the WHO [45] (p. 10), the ADI was “intend[ed] to provide an indicator of safety for over a lifetime of use”, however, the rationale for establishing a PTWI was to demonstrate that metal contaminants “are able to accumulate within the body at a rate and to an extent determined by the level of intake and by the chemical form of the heavy metal present in food. Consequently, the basis on which intake is expressed should be more than the amount corresponding to a single day. Moreover, individual foods may contain above average levels of a heavy metal contaminant, so that consumption of such foods on any particular day greatly enhances that day’s intake” [45] (p. 9). Therefore, it was concluded by the WHO that it was appropriate to establish a weekly, rather than daily, tolerable intake. The WHO emphasized that the ADI and PTWI are intended for application to chronic exposure settings, and for ADI, in particular, it is uncommon for exceedances to occur with short-term exposures, which are seen as easily controllable. In both cases, short-lived exceedances are permissible, as long as they do not persist with monitoring. 

This study compared the WHO RMPPL to the PTWI and found notable differences between them. To illustrate this point, the literature lists both the dietary intake guidelines, but does not recommend one over the other, nor does one guideline seem to supersede the other [45,78,79]. Existing literature, furthermore, does not address conflicting findings, which can cause delay and indecision in developing and utilizing universal guidelines. However, in a more recent publication, the WHO [53] recommends using the PTWI for medicinal plant material metal intake exposures and incorporating the guidelines at the regional and national levels. In the same source, there was no mention or reference to the RMPPL or its status and function. Nevertheless, this may be a first step toward developing universal guidelines. The WHO also discussed the global need to “harmonize” the limits and standards for metals in medicinal plants.

A general literature review reveals a plethora of available dietary guidelines for a suite of metal(loid)s including the WHO RMPPL, the PTWI, the FNB guidelines, and regional and national guidelines for determining metal(loid) contaminants in herbal or medicinal plants. There are differences between the regulations and standards among countries that creates further division and confusion. This was highlighted in the numerous teas studies noted above [28,33,49,54,74,75,76,79] and demonstrates the need to align or conjoin the guidelines. There is also a need to establish standardized, universally accepted concentration levels for metal(loid)s and to identify, develop, and establish guidelines for absent metals (Cs, Th, and U). Tea is consumed worldwide. As such, its utility and distribution necessitates universal guidelines.

## 4. Conclusions

The concentrations of Cd in *T. megapotamicum* tea (0.35 μg/kg) exceeded the WHO RMPPL collectively and in HTAs (0.68 μg/kg). However, calculated human intake did not exceed the RMPPLs set by the WHO or the FNB dietary intake guidelines for As, Cd, Pb, Mo, Se, or V. Similarly, tea sampled from HTAs and LTAs did not exceed the PTWIs or ULs. There was a discrepancy between the WHO RMPPL and the PTWI. However, the PTWI and FNB guideline driven data suggest that the sole consumption of this popular species of tea may not be a dietary risk for metal(loid) ingestion. Again, conflicting and uncertain use of guidelines may be an issue, warranting further development, research, and guidance. As this is a widely used and distributed plant consumed by multiple communities, further research and monitoring are needed to identify factors that affect metal(loid) contamination in *T. megapotamicum* and other herb and medicinal plants used by the Navajo Nation as well as other U mining impacted global communities.

## Figures and Tables

**Table 1:** Metal(loid) concentrations in *T. megapotamicum* plant parts. Data expressed as mean ± SD, reported in mg/kg.

Metal(loid)s	Herb (*n* = 14)	Root (*n* = 14)	Root Soil (*n* = 2)	Topsoil (*n* = 12)	WHO RMPPL mg/kg
**As**	0.423 ± 0.103 ^1,3^	0.758 ± 0.238 ^1^	2.315 ± 1.168	1.844 ± 0.496 ^3^	1
**Cd**	0.346 ± 0.311 ^1,3^	0.634 ± 0.658 ^1^	0.050 ± 0.029	0.512 ± 0.228 ^3^	0.3
**Cs**	0.063 ± 0.074 ^1,4^	0.213 ± 0.169 ^1^	0.488 ± 0.264	1.082 ± 0.357 ^4^	--^5^
**Pb**	0.304 ± 0.731 ^1,3^	0.809 ± 0.286 ^1^	5.783 ± 3.704 ^2^	5.207 ± 1.266 ^2,3^	10
**Mo**	7.916 ± 9.291 ^1,4^	18.304 ± 21.563 ^1^	nd	10.562 ± 5.232 ^4^	--^5^
**Se**	0.738 ± 0.393 ^3^	1.242 ± 0.557	nd	1.122 ± 0.113 ^3^	--^5^
**Th**	0.200 ± 0.248	0.268 ± 0.163	2.642 ± 1.946	2.940 ± 0.815	--^5^
**U**	0.019 ± 0.012 ^1,3^	0.114 ± 0.041^1^	0.829 ± 0.420	1.290 ± 0.224 ^3^	--^5^
**V**	0.244 ± 0.096 ^1,4^	2.343 ± 1.589 ^1^	9.203 ± 4.008	16.297 ± 5.799 ^4^	--^5^

Note: ^1^ Aboveground herb compared to root, *p* < 0.05; ^2^ Root soil compared to topsoil, *p* < 0.05; ^3^ Tea herb compared to topsoil, *p* < 0.05; ^4^ Tea herb compared to topsoil, *p* < 0.001; ^5^ There are no established guidelines in the WHO RMPPL for Cs, Mo, Se, Th, U, or V; nd = not detected.

**Table 2:** Metal(loid) concentrations in aboveground portions of *T. megapotamicum* from HTAs and LTAs. Data expressed as mean ± SD, reported in mg/kg.

Metal(loid)s	High Traffic Areas (HTAs) (*n* = 6)	Low Traffic Areas (LTAs) (*n* = 8)
As	0.360 ± 0.093	0.470 ± 0.086
Cd	0.681 ± 0.106 ^2^	0.095 ± 0.062 ^2^
Cs	0.021 ± 0.008 ^1^	0.095 ± 0.085 ^1^
Pb	0.320 ± 0.030	0.292 ± 0.094
Mo	18.042 ± 3.011 ^2^	0.321 ± 0.171 ^2^
Se	0.996 ± 0.246	0.544 ± 0.379
Th	0.501 ± 0.264	0.087 ± 0.121
U	0.029 ± 0.013 ^1^	0.013 ± 0.005 ^1^
V	0.306 ± 0.119 ^1^	0.198 ± 0.035 ^1^

Note: ^1^ LTAs greater than HTAs, *p* < 0.05; ^2^ HTAs greater than LTAs, *p* < 0.001.

**Table 3:** Summary of dietary exposure to As, Cd, and Pb from the tea consumption of 5 g, 1.54 days per week relative to PTWIs.

Metal(loid)s *	Weekly Intake (μg/kg BW)	PTWI (μg/kg BW)	% below PTWI
As	0.25	15	1.66
Cd	0.20	7	2.85
Pb	0.18	25	0.72

Note: * There is no PTWI for Cs, Mo, Se, Th, U, or V; BW = body weight.

**Table 4:** Summary of the dietary exposure to metal(loid)s from 1.54 days per week of tea consumption relative to the RDI or RDA and UL.

Metal(loid)s *	Daily Intake (μg)	RDI or RDA and UL (μg/day)	% below RDI or RDA and UL
Mo	8.71	RDA: 45	19.35
		UL: 2000	0.44
Se	0.81	RDI: 55	1.47
		UL: 400	0.20
V	0.27	UL: 1800	0.02

Note: * There are no RDIs, RDAs or ULs for As, Cd, Cs, Pb, Th, or U.

**Table 5:** Summary of dietary exposure to As, Cd, and Pb from a 5 g intake of tea 1.54 days per week comparing the PTWIs of HTAs to LTAs.

Metal(loid)s in HTAs and LTAs	Weekly Intake μg/kg BW	PTWI (μg/kg BW)	% Below PTWI
HTA As	0.21		1.4
LTA As	0.27	15	1.8
HTA Cd	0.40		5.71
LTA Cd	0.06	7	0.86
HTA Pb	0.19		0.76
LTA Pb	0.17	25	0.68

**Table 6:** Summary of the dietary exposure to metal(loid)s from tea consumption representative of 1.54 days per week in comparison to the RDI or RDA, and UL in HTA and LTA.

Metal(loid)s in HTAs and LTAs	Daily Intake (μg)	RDI or RDA and UL (μg/day)	% below RDI or RDA and UL
HTA Mo	19.85	RDA: 45; UL: 2000	44.1; 0.99
LTA Mo	0.4		0.88; 0.02
HTA Se	1.10	RDI: 55; UL: 400	2.0; 0.3
LTA Se	0.61		1.11; 0.15
HTA V	0.34	UL: 1800	0.02
LTA V	0.22		0.01
